# Effective Force Generation During Mammalian Cell Migration Under Different Molecular and Physical Mechanisms

**DOI:** 10.3389/fcell.2022.903234

**Published:** 2022-05-19

**Authors:** Lingxing Yao, Yizeng Li

**Affiliations:** ^1^ Department of Mathematics, University of Akron, Akron, OH, United States; ^2^ Department of Mechanical Engineering, Kennesaw State University, Marietta, GA, United States

**Keywords:** cell migration, biphasic response, force generation, mechanosensitivity, energy generation

## Abstract

We have developed much understanding of actin-driven cell migration and the forces that propel cell motility. However, fewer studies focused on estimating the effective forces generated by migrating cells. Since cells *in vivo* are exposed to complex physical environments with various barriers, understanding the forces generated by cells will provide insights into how cells manage to navigate challenging environments. In this work, we use theoretical models to discuss actin-driven and water-driven cell migration and the effect of cell shapes on force generation. The results show that the effective force generated by actin-driven cell migration is proportional to the rate of actin polymerization and the strength of focal adhesion; the energy source comes from the actin polymerization against the actin network pressure. The effective force generated by water-driven cell migration is proportional to the rate of active solute flux and the coefficient of external hydraulic resistance; the energy sources come from active solute pumping against the solute concentration gradient. The model further predicts that the actin network distribution is mechanosensitive and the presence of globular actin helps to establish a biphasic cell velocity in the strength of focal adhesion. The cell velocity and effective force generation also depend on the cell shape through the intracellular actin flow field.

## 1 Introduction

Mammalian cells under different biophysical environments use diverse mechanisms to migrate. For example, when cells spread on a two-dimensional substrate, cell migration relies on actin polymerization and myosin contraction (Gardel et al., 2010; Murrell et al., 2015). When cells are confined in one-dimensional space, water permeation through the cell membrane can drive cell migration (Stroka et al., 2014; Tao et al., 2017; Li and Sun 2018; Li et al., 2019; Li et al., 2020). When cells reside in three-dimensional collagen matrices, various migration modes can occur, including bleb-based, lamellipodia-based, and lobopodia-based modalities (Petrie and Yamada 2016). The mechanisms of force generation are different for different cell migration modes, but the details are not well studied.

The force generation of actin-driven migration has been studied for a long time. Both modeling and experimental works have quantified the effective force from actin polymerization (Mogilner and Oster, 1996; Mogilner and Oster, 2003; Carlsson, 2003; Dickinson et al., 2004; Parekh et al., 2005; Prass et al., 2006). Traction force microscopy, on the other hand, examines how much force is delivered by cells onto the substrate (Style et al., 2014). Compared to actin-driven cell migration, the force generated from other migration mechanisms is less investigated (Petrie and Yamada, 2015; Li et al., 2019). In this paper, we use a multi-modular theoretical model to compare and discuss the mechanisms of force generation under actin-driven and water-driven cell migration. This model is a further development from our early works (Li and Sun, 2018; Li et al., 2019) by including the globular actin (G-actin) phase and the interconversion between G-actin and filamentous actin (F-actin). The new model provides insights into the biphasic cell velocity and mechanosensitivity, together with cell force generation and energy output, which have not been adequately studied.

We will use free energy identities to determine the biomolecular and biophysical processes responsible for cell energy generation. In quantifying force generation, instead of calculating the forces produced by the molecules, we determine the effective forces generated by entire cells onto the environment during cell migration. This calculation enables us to quantify how much resistive force a cell can sustain from the physical environment while maintaining its migration. The results have implications on *in vivo* cell migration where the environment often presents challenging conditions on cells. The model prediction will also be compared to existing experimental data whenever available.

## 2 Modeling Methods

One of the main innovations of this work is the inclusion of G-actin and its interaction with the rest of the system in determining force generation. To provide sufficient technical background for later discussion and analysis, we begin with a detailed description of the entire model. The multi-modular model contains three modules: cytosol, actin, and solute. Including these modules are essential in studying cell force generation in different environments. The cytosol and extracellular fluid constitute the fluid environment of the cell. The cytosol is a continuous water-like Newtonian fluid (Keren et al., 2009) which exists both inside and outside of the cell. When the extracellular fluid flows into the cell via aquaporins and membrane diffusion, the extracellular fluid converts into the cytosol and vice versa. For this reason, we will use cytosol to refer to the water-like fluid in the entire computational domain. The actin module provides one of the well-known mechanisms of cell migration driven by actin polymerization. In this work, we explicitly include G-actin and consider the interchange of F-actin and G-actin. This inclusion will enable us to study actin dynamics that is missing in models that contain F-actin only (Li and Sun, 2018; Li et al., 2019). The solute is a collection of ions and small molecules. This module studies the variation of the intracellular osmotic pressure under different conditions and provides the physics basis of water flux. The solute can diffuse within the cell and be transported across the cell membrane. The membrane has various passive channels and active pumps. In addition to the solute transportation, we will consider the contribution of the channels and pumps to the force generation of cells. The fluid-structure interaction and the coupling of the three modules will be described in each subsection below.

We use Ω to denote the cell domain and *∂*Ω to indicate the boundary of the cell, i.e., cell membrane. *∂*Ω^−^ is the interior side of the cell membrane, and *∂*Ω^+^ indicates the exterior side. In the two-dimensional implementation, the moving boundary problem is solved by the Immersed Boundary Method (Peskin, 2002). In the one-dimensional implementation, the domain Ω reduces to *x* ∈ [*x* ^b^, *x* ^f^], where *x* ^b^(*t*) and *x* ^f^(*t*) are, respectively, the back and front positions of the cell. We will use subscript “f” to indicate quantities associated with the front of the cell and “b” for the back. Under steady-state, *x* ^f^(*t*) − *x* ^b^(*t*) ≡ *L* is the constant cell length. In this case, we use *x* ∈ [0, *L*] to describe the computational domain in the moving frame of the migrating cell.

### 2.1 Cytosol Module

The fluid motion within and surrounding mammalian tissue cells is of low Reynolds number and non-compressible. We thus neglect the inertia and non-linear terms in its momentum equation. The conservation of momentum and mass of the cytosol is

$$-\nabla p+\mu \nabla^{2}v_{c}-\eta \theta_{n}\left(v_{c}-v_{n}\right)=0,\;\nabla \cdot v_{c}=0,$$ (1)

where ***v***_*c*_, *p*, and *μ* are the velocity, hydrostatic pressure, and dynamic viscosity of the cytosol, respectively; ***v***_*n*_ is the velocity of the F-actin network; *θ*_*n*_ is the concentration of actin molecule in the filamentous form; and *η* is the coefficient of interfacial friction between the F-actin-network and the cytosol. The interfacial stress term, *ηθ*_*n*_ (***v***_*c*_ − ***v***_*n*_), comes from the velocity difference between the actin and cytosol phases. Under mean-field approximation, this stress serves as an effective body force on the cytosol. If the interfacial stress is much larger than the viscous shear stress, then the viscous term can be neglected.

The flux boundary condition for cytosol should satisfy the continuity of its velocity across the membrane in the normal direction and non-slip condition along the membrane in the tangential direction, i.e.,

$$v_{c}-\frac{\partial X}{\partial t}=-J_{\text{water}}n,\;\text{on }\partial \Omega ,$$ (2)

where ***n*** is the outward norm on the cell membrane, **X** is the membrane position, and *J*_water_ is the water flux across the cell membrane. The water flux is determined by the difference of the solute concentration and the hydrostatic pressure difference across the cell membrane, i.e.,

$$J_{\text{water}}=-\alpha_{\text{w}}\left(\psi {|}_{\partial \Omega^{-}}-\psi {|}_{\partial \Omega^{+}}\right),\;\psi =p-RTc,$$ (3)

where *α*_w_ is the permeability of water, which depends on the expression of aquaporins. *c* is the solute concentration, and *RT* is the ideal gas constant times the absolute temperature. *ψ* is the approximated chemical potential of water for low solute concentration (less than 1 M). Such defined water flux is positive if it flows into the cell and negative otherwise.

The difference of the cytosol pressure, *p*, across the cell membrane balances the stress in the F-actin network, *σ*, and the stress in the cell membrane, **F**_*m*_, i.e.,

$$\left(p{|}_{\partial \Omega^{-}}-p{|}_{\partial \Omega^{+}}\right)n=\sigma n+F_{m}{\left|\frac{\partial X}{\partial s}\right|}^{-1},\;F_{m}=k_{m}\frac{\partial }{\partial s}\left(\left|\frac{\partial X}{\partial s}-\frac{\partial X_{r}}{\partial s}\right|\frac{\partial X/\partial s}{\left|\partial X/\partial s\right|}\right),$$ (4)

where *k*_*m*_ is the membrane modulus, s∈R/(2πZ) is the material coordinate on the cell membrane, and **X**_*r*_ describes the membrane shape at *t* = 0. In the two-dimensional implementation, the extracellular fluid velocity and pressure are numerically computed. In the one-dimensional implementation, the extracellular fluid field is analytically solved through a pipe flow model. We use *d*_*g*_ to represent the coefficient of hydraulic resistance, which is affected by the extracellular geometry, fluid viscosity, and permeability if a porous matrix is considered (Li and Sun 2018; Maity et al., 2019). The hydraulic pressure acting on the cell from the extracellular domain is determined by the coefficient of hydraulic resistance, cell velocity, and water flux. Under steady states, this pressure is solved by

$$p{|}_{L^{+}}=p_{0}^{\text{f}}+d_{g}^{\text{f}}\left(v_{0}-J_{\text{water}}^{\text{f}}\right),\;p{|}_{0^{-}}=p_{0}^{\text{b}}-d_{g}^{\text{b}}\left(v_{0}-J_{\text{water}}^{\text{b}}\right),$$ (5)

where *v*_0_ is the steady-state cell velocity and *p*_0_ is the ambient hydrostatic pressure at infinity. We enforce $J_{\text{water}}^{\text{b}}=-J_{\text{water}}^{\text{f}}$ so that the total cytosol content is conserved under steady-state.

### 2.2 Actin Module

The F-actin network contains myosin, which contracts the network. In this work, we do not explicitly model the myosin molecules but treat the myosin contraction as a parameter. The network stress can be decomposed into two components: a passive part comes from the actin filament swelling, *σ*_*n*_, and an active part comes from myosin contraction, *σ*_*a*_, i.e., *σ* = *σ*_*n*_ − *σ*_*a*_. We use a linear constitutive approximation for the actin filament swelling, i.e., $\sigma_{n}=k_{\sigma_{n}}\theta_{n}$ , where $k_{\sigma_{n}}$ is the coefficient of F-actin pressure. The active contraction from myosin, *σ*_*a*_, is taken as a parameter. The F-actin network connects to the substrate through focal adhesion which exerts stress on the actin network as the actin flows. We model the stress from focal adhesion as an effective body force on the network proportional to the velocity of the actin network. This body force is balanced by the stress gradient in the actin network and the interfacial stress between the actin and cytosol. Therefore, the force balance equation of the F-actin network is

$$-\nabla \sigma +\eta \theta_{n}\left(v_{c}-v_{n}\right)-\eta_{\text{s}t}\theta_{n}v_{n}=0,$$ (6)

where *η*_s*t*_ is the strength of focal adhesion, the value of which depends on the extracellular mechanical and biochemical properties and the geometry of the space (Liu et al., 2015; Paluch et al., 2016). In Eq. 6, the first two terms are the internal forces come within the cell, whereas the third term is the external force comes from the substrate (environment).

The modeling of mass conservation of F-actin and the process of (de)polymerization depends on the choice of the molecular details. In our early models, G-actin was not included (Li and Sun, 2018; Li et al., 2019). Below we begin with briefly reviewing the model without G-actin, followed by a new model with G-actin.

#### 2.2.1 A Model Without G-Actin

In our early model (Li et al., 2019), in the absence of G-actin, F-actin polymerization and depolymerization happen at the front and back ends of the cell, respectively, and there is no reaction of actin in the interior of the cell. In this case, the mass conservation of the F-actin is

$$\frac{\partial \theta_{n}}{\partial t}+\nabla \cdot \left(v_{n}\theta_{n}\right)=0.$$ (7)

At the cell boundary, the actin flux should be consistent with the amount of actin that are added or removed,

$$\theta_{n}\left(\frac{\partial X}{\partial t}-v_{n}\right)\cdot n=J_{\text{actin}},\;\text{on }\partial \Omega ,$$ (8)

where *J*_actin_ is the rate of actin (de)polymerization prescribed on the cell boundary. It is non-zero at the front and back regions of the cell where actin polymerization or depolymerization exists and is zero elsewhere. We enforce *∫*_*∂*Ω_ *J*_actin_ *ds* = 0 around the cell boundary to conserve the total F-actin within the cell. In the one-dimensional implementation, this condition reduces to $J_{\text{actin}}^{\text{b}}=-J_{\text{actin}}^{\text{f}}$ . The average concentration of actin molecules in the filamentous form, *θ*_*n*,*_, can be incorporated in the initial condition in a transient model or by enforcing *∫*_Ω_ *θ*_*n*_ *dV* = *V*_Ω_ *θ*_*n*,∗_ in a steady-state model, where *V*_Ω_ is the volume of domain Ω.

#### 2.2.2 A Model With G-Actin

In the new model developed in this work, we explicitly include G-actin and the interplay of F-actin and G-actin. We still let actin polymerization happen at the front of the cell (Ridley, 2011), but depolymerization occurs throughout the cytoplasm. Actin polymerization consumes G-actin, whereas depolymerization converts F-actin into G-actin. In this case, the mass conservation of F-actin is

$$\frac{\partial \theta_{n}}{\partial t}+\nabla \cdot \left(v_{n}\theta_{n}\right)=-\gamma \theta_{n},$$ (9)

where *γ* is the rate of actin depolymerization. The flux boundary condition for *θ*_*n*_ is the same as Eq. 8 except that *J*_actin_ is only non-zero at the front region of the cell where actin polymerization happens and is zero elsewhere. G-actin diffuses in the cytosol and is also convected by the cytosol flow. The diffusion-advection-reaction equation for G-actin is

$$\frac{\partial \theta_{c}}{\partial t}+\nabla \cdot \left(v_{c}\theta_{c}\right)=\nabla \cdot \left(D_{\theta_{c}}\nabla \theta_{c}\right)+\gamma \theta_{n},$$ (10)

where *θ*_*c*_ and $D_{\theta_{c}}$ are, respectively, the concentration and diffusion coefficient of G-actin within cytosol. The flux boundary condition for G-actin is determined by the rate of actin polymerization at the front of the cell, i.e.,

$$\theta_{c}\left(\frac{\partial X}{\partial t}-v_{c}\right)\cdot n=-J_{\text{actin}},\;\text{on }\partial \Omega .$$ (11)

Since the newly polymerized F-actin is converted from G-actin, the rate of actin polymerization, *J*_actin_, can be modeled as a function of G-actin concentration by $J_{\text{actin}}^{\text{f}}=J_{\text{actin},0}^{\text{f}}\theta_{c}/\left(\theta_{c,c}+\theta_{c}\right)$ , where $J_{\text{actin},0}^{\text{f}}$ is the coefficient of actin polymerization and *θ*_*c*,*c*_ is a constant scaled by the critical concentration of G-actin (Pollard et al., 2000). The combination of Eqs 9 and 10 and their boundary conditions ensure the conservation of the total amount of the actin molecules, which means that *∫*_Ω_(*θ*_*n*_ + *θ*_*c*_)*dV* = *V*_Ω_ *θ*_∗_ is satisfied, where θ_*_ is the average actin concentration. The rate of actin depolymerization, *γ*, modulates the average concentration of F-actin and G-actin: high *γ* leads to a high average concentration of G-actin because most F-actin depolymerizes into G-actin. To obtain a controlled comparison of the results from models with and without G-actin, we adjust *γ* such that the average concentration of F-actin in the model with G-actin is the same as that without G-actin.

### 2.3 Solute Module

In the solute module, we lump all charged ions and small molecules into a single species of electro-neutral solute. This lumped model is adequate in studying osmosis and water flux (Jiang and Sun, 2013; Stroka et al., 2014). An ion-specific model can also be included if individual ion concentrations, channels, transporters, and pumps are studied (Li et al., 2015; Yellin et al., 2018; Li et al., 2021). The diffusion-advection equation for solute is

$$\frac{\partial c}{\partial t}+\nabla \cdot \left(v_{c}c\right)=\nabla \cdot \left(D_{c}\nabla c\right),$$ (12)

where *c* is the concentration of solution and *D*_*c*_ is its diffusion coefficient. At the cell boundary, the solute can be transported in and out of the cell through both passive channels and active pumps. The passive flux is proportional to the chemical potential difference of the solute across the membrane. Since the intracellular and extracellular osmosis only differ by ∼0.1% (Li et al., 2021), the passive flux can be approximated by a first-order Taylor expansion of the chemical potential difference, i.e.,

$$J_{c,p}=-k_{\text{sol}}\left[\left(c{|}_{\partial \Omega^{-}}\right)-\left(c{|}_{\partial \Omega^{+}}\right)\right],$$ (13)

where *k*_sol_ is the permeability coefficient of solute. The minus sign comes from our convention that all fluxes are positive when they flow into the cell and the direction of the fluxes is always normal to the cell membrane. The active flux, *J*_*c*,active_, is controlled by active pumps and secondary transporters (Gadsby, 2009). The expression level of pumps and transporters are cell-type dependent and is also affected by biophysical processes in the cell. In this work, we treat these processes as known and prescribe the active flux as parameters.

Taken together, the flux boundary condition for solute is

$$\left(v_{c}c-D_{c}\nabla c\right)\cdot n=c\frac{\partial X}{\partial t}\cdot n-J_{c,p}-J_{c,\text{active}},\;\text{on }\partial \Omega .$$ (14)

In the model, the passive flux exists everywhere on the boundary whereas the active flux is only prescribed at the front and back regions of the cell. In the one-dimensional implementation, without loss of generality, $J_{c,\text{active}}^{\text{b}}=-J_{c,\text{active}}^{\text{f}}$ is used to model polarized distribution of active pumps. This anti-symmetric active solute flux is a generic modeling choice, not a requirement of conservation laws. Under steady state the conservation law should be applied to the total flux, i.e., $J_{c,p}^{\text{b}}+J_{c,\text{active}}^{\text{b}}=-J_{c,p}^{\text{f}}-J_{c,\text{active}}^{\text{f}}$ .

### 2.4 Forces on the Cell

For a migrating cell, in addition to focal adhesion, the cell membrane has mechanical interaction with the substrate or matrix through friction and other adhesive molecules. We lump all resistive forces together into an effective adhesive force that is proportional to the velocity of the cell membrane, i.e., $F_{\text{ad}}=-k_{\text{ad}}\left(\left(\partial X/\partial t\right)\cdot n\right)n$ , where *k*_a*d*_ is the coefficient of adhesion. Migrating cells may also experience physical obstacles, such as proteins or fibers in the extracellular cellular matrix. We use an external force normal to the cell surface to represent the effects of the obstacles, i.e., − ***f***_ext_ ***n***. This force only applies to the front region of the cell.

In the one-dimensional implementation, when cells reach a steady state at velocity *v*_0_, the adhesive force reduces to $F_{\text{a}d}^{\text{b}}=-k_{\text{a}d}v_{0}$ applied to the back of the cell. The external force becomes a normal force at the front of the cell that works against the direction of cell migration. By taking the cell membrane, cytosol, actin, and myosin as a whole system, we can write all the forces acting on the system. These forces consist of the extracellular hydrostatic pressure on the two ends of the cell, the effective body force on the actin network from the focal adhesion, the adhesive force at the back of the cell, and the external force from the environment. Under a steady-state, these forces should sum up to zero to provide a force balance condition for a one-dimensional, i.e.,

$$\left(p{|}_{0^{-}}\right)-\left(p{|}_{L^{+}}\right)-\int_{0}^{L}\eta_{\text{st}}\theta_{n}v_{n}dx+F_{\text{ad}}^{\text{b}}-f_{\text{ext}}^{\text{f}}=0.$$ (15)

### 2.5 Parameters

Two of the most critical parameters in the model are the rate of actin polymerization and the rate of active fluxes. These two parameters control the driving force of the actin-driven and water-driven cell migration (Li and Sun, 2018). Below we use the one-dimensional implementation to discuss the choice of the two parameters.

The boundary condition of the F-actin flux (Eq. 8) indicates that the rate of actin polymerization affects the velocity of actin retrograde flow in the absence of cell membrane motion. Experimental observations show that the velocity of actin retrograde flow can vary from about 20 nm/s up to the order of 100 nm/s (Kiuchi et al., 2007; Gardel et al., 2008; Vitriol et al., 2015). On the other hand, the average concentration of actin molecules in the filamentous form is about 200 μM (the value of *θ*_*n*,*_ in the model) (Satcher and Dewey, 1996; Pollard et al., 2000). Therefore, the rate of actin polymerization at the front of the cell is estimated as *J*_actin_ = 6 nm⋅mM/s. In the early model without G-actin, where polymerization and depolymerization happen at the two ends of the cell, we let $J_{\text{actin}}^{\text{f}}=-J_{\text{actin}}^{\text{b}}=6$ nm⋅mM/s as the input parameter. In this new model with G-actin, where actin polymerization happens at the front end and depolymerization happens throughout the cytoplasm, the average concentration of actin in combined globular and filamentous forms is taken as θ_*_ = 400 μM (Satcher and Dewey, 1996; Pollard et al., 2000) and the concentration constant in polymerization is *θ*_*c*,*c*_ = 0.2 μM (Pollard et al., 2000). We therefore let $J_{\text{actin},0}^{\text{f}}=10$ mM⋅nm/s, which provides the maximum rate of actin polymerization.

The active solute flux is zero unless water-driven cell migration is studied. The flux through individual ion pumps is on the other of 10^4^ per second (Gadsby, 2009). Depending on cell types and pump species, the number of active pumps is on the order of 10^2^ to 10^3^ per μm^2^ (Landowne and Ritchie, 1970; Baker and Willis, 1972; Burnham and Stirling, 1984a; Burnham and Stirling, 1984b). Together, we expect 10^6^ to 10^7^ solute particles to be pumped across the cell membrane per μm^2^ per second. We therefore let the parameter for the active pump flux to be $J_{c,\text{active}}^{\text{f}}=-J_{c,\text{active}}^{\text{b}}=16\;\mu$ m mM/s. This flux corresponds to $∼10^{7}$ solute particles per μm^2^ per second. In addition, this value of flux generates the same maximum cell velocity as the actin-driven case so that we can compare the two mechanisms of cell migration together.

The rest of the parameters used in the model are provided in Table 1. The listed coefficient of focal adhesion strength, *η*_st_, is based on the experimental measurement of the relation between traction force and the velocity of actin flow (Gardel et al., 2008). We use this value when computing actin-driven cell migration, which typically happens in open spaces. When cells are in confinement, cell migration has reduced or minimal dependence on focal adhesion (Liu et al., 2015; Paluch et al., 2016). Since water-driven cell migration typically happens in confinements (Stroka et al., 2014), we use a two-order of magnitude lower *η*_st_ when water-driven cell migration is studied. This lower value is also used in the two-dimensional implementation to ensure numerical stability. To compensate for the reduced focal adhesion strength, we use *J*_actin_ = 150 nm/s/mM in the two-dimensional implementation to maintain a similar output of cell velocity.

**Table 1:** Parameters used in the one-dimensional model. These are the default parameters unless otherwise specified.

Parameters	Description	Values	Sources
*R* (J/mol/K)	Ideal gas constant	8.31	Physical constant
*T* (K)	Absolute temperature	310	Cell environment
*L* (μm)	Cell length in 1D	50	Mistriotis et al. (2019)
*r* (μm)	Cell radius in 2D	14.92	Typical cell size
*μ* (Pa⋅s)	Dynamic viscosity of cytosol	2 × 10^–3^	Similar to water
*η* (Pa⋅s/μm^2^/mM)	Drag coefficient between two phases	10^–2^	Dembo and Harlow (1986)
*η*_st_ (Pa⋅s/μm^2^/mM)	Coefficient of drag from focal adhesion	10^3^	Gardel et al. (2008)
$k_{\sigma_{n}}$ (Pa/mM)	Coefficient for the passive F-actin stress	10^3^	Estimated
*k*_ad_ (Pa⋅s/μm)	Coefficient in the adhesive force	600	Estimated
*k*_*m*_ (Pa)	Membrane modulus	0.2	Estimated
*d*_*g*_ (Pa⋅s/μm)	Coefficient of hydraulic pressure	10^–2^	Stroka et al. (2014)
*D*_*c*_ (μm^2^/s)	Diffusion coefficient of solute	1	Stroka et al. (2014)
$D_{\theta_{c}}$ (μm^2^/s)	Diffusion coefficient of G-actin	1	Estimated
$k_{\text{sol}}^{\text{f}}$ (μm/s)	Passive channel coefficient at the front	50	Estimated
$k_{\text{sol}}^{\text{b}}$ (μm/s)	Passive channel coefficient at the back	50	Estimated
$\alpha_{\text{w}}^{\text{f, b}}$ (μm/Pa/s)	Water permeability constant	10^–4^	Stroka et al. (2014)
$p_{0}^{\text{f, b}}$ (Pa)	Extracellular hydraulic pressure	0	Free parameter
$c_{0}^{\text{f, b}}$ (mM)	Extracellular solute concentration	340	Stroka et al. (2014)

## 3 Model Analysis and Validation

**FIGURE 1:**
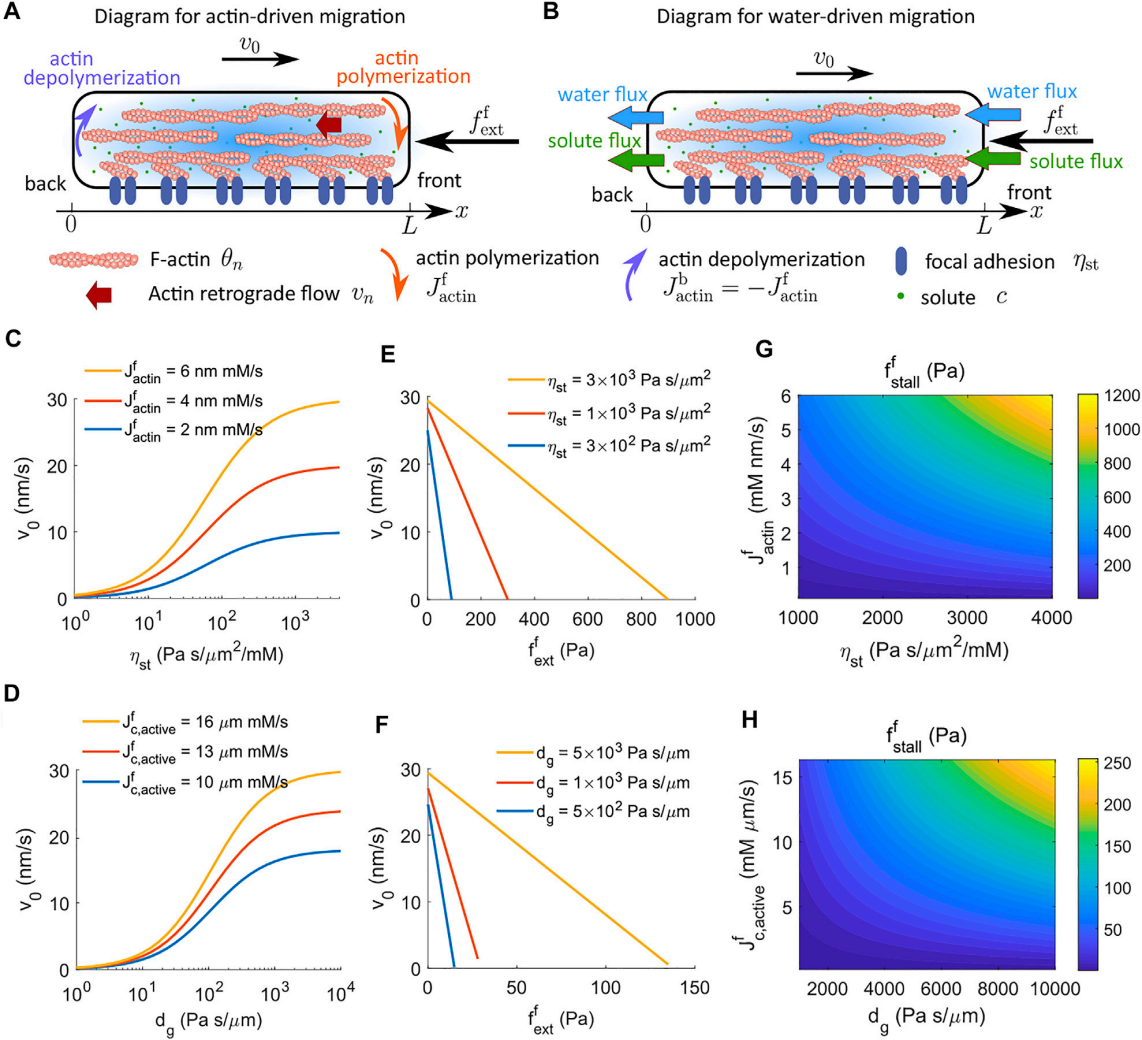
Cell velocity and effective force generation under actin-driven and water-driven cell migration in the absence of G-actin. **(A)** Schematics of actin-driven cell migration where actin polymerization and depolymerization happen at the front and back ends of the cell, respectively. G-actin is not explicitly modeled. **(B)** Schematics of water-driven cell migration. **(C)** Model prediction of the velocity of actin-driven cell migration as a function of the strength of focal adhesion, *η*_st_, for different rates of actin polymerization at the front end of the cell. **(D)** Model prediction of the velocity of water-driven cell migration as a function of the coefficient of external hydraulic resistance, *d*_*g*_, for different rates of actin polymerization at the front end of the cell. **(E)** Predicted actin-driven cell velocity as a function of the external force for different strengths of focal adhesion. **(F)** Predicted water-driven cell velocity as a function of the external force for different coefficients of external hydraulic resistance. **(G)** The contour of the stall force per unit area for actin-driven cell migration as a function of the rate of actin polymerization and the strength of focal adhesion. **(H)** The contour of the stall force per unit area for water-driven cell migration as a function of the active solute flux and the coefficient of external hydraulic resistance.

We begin by recapitulating the linear analysis of the one-dimensional implementation of steady-state cell migration in the absence of G-actin (Section 2.2.1; Figure 1A). The analysis will provide insight into the biophysical mechanisms behind cell migration in different environments. It also serves as a foundation for later model prediction and interpretation when we consider the presence of G-actin or cell shapes. We will validate the model through quantitative comparison with existing experimental data.

### 3.1 Linear Analysis on Actin- and Water-Driven Cell Migration

Our model permits a closed-form analytical solution expressed in terms of the driving mechanisms. Integrating the sum of the cytosol force balance equation (Eq. 1) and the F-actin force balance equation (Eq. 6), along with the corresponding boundary conditions, leads to an instructive expression of the steady-state cell velocity,

$$v_{0}=\frac{\eta_{\text{st}}L}{\eta_{\text{st}}L\theta_{n,*}+d_{g}+k_{\text{ad}}}J_{\text{actin}}^{\text{f}}+\frac{d_{g}}{\eta_{\text{st}}L\theta_{n,*}+d_{g}+k_{\text{ad}}}J_{\text{water}}^{\text{f}}.$$ (16)

The first term in the equation represents the contribution from actin-driven cell migration where actin polymerization, *J*_actin_, is the driving agent (Figure 1A). The second term in the equation represents the contribution from water-driven cell migration where directional water flux, *J*_water_, is the apparent driving agent (Figure 1B). While the rate of actin polymerization is a prescribed parameter in the model without G-actin, the water flux is not given but computed by Eq. 3. Polarized active flux is the fundamental cause of the directional water flux (Stroka et al., 2014; Li and Sun, 2018).

Equation 16 suggests that (*η*_st_ *Lθ*_*n*,∗_ + *d*_*g*_ + *k*_ad_) services as the effective resistance of cell migration. Increasing the strength of focal adhesion, *η*_st_, increases the velocity of actin-driven cell migration (Figure 1C), whereas increasing the coefficient of extracellular hydraulic resistance, *d*_*g*_, increases the velocity of water-driven cell migration (Figure 1D). Of note, the model without G-actin predicts that the actin-driven cell velocity increases monotonically with the strength of focal adhesion (Figure 1C). In experiments, cells were found to show biphasic response in the strength of focal adhesion, i.e., excessive strong focal adhesion leads to a reduction in cell velocity (DiMilla et al., 1993; Palecek et al., 1997; Gupton and Waterman-Storer 2006; Gardel et al., 2008; Kim and Wirtz, 2013). We will discuss this in Section 4.2 where G-actin is included.

We can perform linear analysis on the model by assuming linear profiles of the intracellular F-actin and solute concentrations. The water flux across the cell membrane is thus approximated by

$$J_{\text{water}}^{\text{f}}=-J_{\text{water}}^{\text{b}}=≃\frac{\alpha_{\text{w}}d_{g}v_{0}+\alpha_{\text{w}}\eta LJ_{\text{actin}}^{\text{f}}+\alpha_{\text{w}}\eta_{\text{sol}}LJ_{c,\text{active}}^{\text{f}}}{2+\alpha_{\text{w}}d_{g}+\alpha_{\text{w}}L\left(\eta \theta_{n,*}+\eta_{\text{sol}}c_{0}\right)},$$ (17)

where *c*_0_ is the constant extracellular solute concentration and

$$\eta_{\text{sol}}=\frac{RT}{D_{c}+k_{\text{sol}}L/2}$$ (18)

is the effective coefficient of reactive force from active pumps. Equation 17 suggests that the cell velocity, actin polymerization, and active solute flux contribute to the water flux across the cell membrane via, respectively, the hydraulic resistance, *d*_*g*_, interfacial friction, *η*, and solute transportation and diffusion, *η*_sol_. Substituting Eq. 17 into Eq. 16 provides a relation between the cell velocity and the fundamental driving forces,

$$\begin{array}{ll} v_{0} & ≃\frac{1}{K_{c}}\left[\left(\eta_{\text{st}}L+\frac{\alpha_{\text{w}}d_{g}\eta L}{2+\alpha_{\text{w}}d_{g}+\alpha_{\text{w}}L\left(\eta \theta_{n,*}+\eta_{\text{sol}}c_{0}\right)}\right)J_{\text{actin}}^{\text{f}}\right.\\ & \left.\;+\frac{\alpha_{\text{w}}d_{g}\eta_{\text{sol}}L}{2+\alpha_{\text{w}}d_{g}+\alpha_{\text{w}}L\left(\eta \theta_{n,*}+\eta_{\text{sol}}c_{0}\right)}J_{c,\text{active}}^{\text{f}}\right], \end{array}$$ (19)

where

$$K_{c}=\left(\eta_{\text{st}}L\theta_{n,*}+d_{g}+k_{\text{ad}}\right)-\frac{\alpha_{\text{w}}d_{g}^{2}}{2+\alpha_{\text{w}}d_{g}+\alpha_{\text{w}}L\left(\eta \theta_{n,*}+\eta_{\text{sol}}c_{0}\right)}$$

is the effective resistance of cell migration. The product of water permeability and hydraulic resistance, *α*_w_ *d*_*g*_, is a dimensionless number indicating the importance of fluid dynamics in cell migration. When *α*_w_ *d*_*g*_ ≪ 1, Eq. 19 can be simplified as

$$v_{0}≃\frac{1}{K_{c}}\left[\left(\eta_{\text{st}}L\right)J_{\text{actin}}^{\text{f}}+\frac{\left(\alpha_{\text{w}}d_{g}\right)\left(\eta_{\text{sol}}L\right)}{2+\alpha_{\text{w}}d_{g}+\alpha_{\text{w}}L\left(\eta \theta_{n,*}+\eta_{\text{sol}}c_{0}\right)}J_{c,\text{active}}^{\text{f}}\right].$$ (20)

The dropped term is the contribution of water flux to actin-driven cell migration in the absence of active solute flux. This term is negligible for small hydraulic resistance and will play a role for large *α*_w_ *d*_*g*_, which we will discuss later.

Equation 20 provides several interesting biophysical insights into actin- and water-driven cell migration. The term $\eta_{\text{sol}}J_{c,\text{active}}^{\text{f}}$ in water-driven mechanism is analogous to the term $\eta_{\text{st}}J_{\text{actin}}^{\text{f}}$ in actin-driven mechanism. *η*_st_ from focal adhesion generates the reactive forces when actin polymerization happens, whereas *η*_sol_ from transportation and diffusion generates the reactive forces when active solute pumping happens.

### 3.2 Analysis on the Factors That Affect the Force Generated by a Migrating Cell

Cells *in vivo* constantly interact with complex environments with various physical barriers. Understanding the effective force generated by cells will provide insights into how cells manage to overcome challenging environments. To achieve this goal, we use our model to quantify the magnitude of force or pressure needed at the front of the cell to stall cell migration. This approach is different from techniques such as traction force microscopy (Style et al., 2014) that enable us to estimate the stress passes from cells onto the substrate.

We apply an external force per unit area, $f_{\text{ext}}^{\text{f}}$ , i.e., an external pressure, to the front of the cell (Figures 1A,B). The direction of the force is defined positive when it hinders cell migration. In the presence of the external force, Eq. 20 becomes

$$v_{0}≃\frac{1}{K_{c}}\left[\left(\eta_{\text{st}}L\right)J_{\text{actin}}^{\text{f}}+\frac{\left(\alpha_{\text{w}}d_{g}\right)\left(\eta_{\text{sol}}L\right)}{2+\alpha_{\text{w}}d_{g}+\alpha_{\text{w}}L\left(\eta \theta_{n,*}+\eta_{\text{sol}}c_{0}\right)}J_{c,\text{active}}^{\text{f}}-f_{\text{ext}}^{\text{f}}\right].$$ (21)

The cell velocity is predicted to decrease linearly with increasing external force (Figures 1E,F).

Stall force is defined as the external force that stalls cell migration. The model predicts that the stall force per unit area increases with the strength of focal adhesion, *η*_st_, for actin-driven cell migration (Figure 1E) and increases with the coefficient of external hydraulic resistance, *d*_*g*_, for water-driven cell migration (Figure 1F). In addition to the strength of focal adhesion or hydraulic resistance, the stall force depends on multiple factors at the molecular level. We can solve for the stall force from Eq. 21 when the cell velocity vanishes, i.e.,

$$f_{\text{stall}}^{\text{f}}=\left(\eta_{\text{st}}L\right)J_{\text{actin}}^{\text{f}}+\frac{\left(\alpha_{\text{w}}d_{g}\right)\left(\eta_{\text{sol}}L\right)}{2+\alpha_{\text{w}}d_{g}+\alpha_{\text{w}}L\left(\eta \theta_{n,*}+\eta_{\text{sol}}c_{0}\right)}J_{c,\text{active}}^{\text{f}}.$$ (22)

For actin-driven cell migration, i.e., without active solute flux, Eq. 22 indicates that the stall force per unit area is the product of $\left(\eta_{\text{st}}L\right)J_{\text{actin}}^{\text{f}}$ . Figure 1G shows the contour of the stall pressure as an increasing function of the rate of actin polymerization, $J_{\text{actin}}^{\text{f}}$ , and the strength of focal adhesion, *η*_st_. The predicted stall pressure is on the order of 1 kPa for a physiologically relevant rate of actin polymerization and strength of focal adhesion. An average cell has cross-sectional areas of 30–50 μm^2^. Hence, the effective stall force for actin-driven cell migration is predicted to be on the order of 30–50 nN, which is consistent with experimental measurement (Oliver et al., 1995; Prass et al., 2006).

For water-driven cell migration, i.e., without actin polymerization, Eq. 22 indicates that the stall force per unit area depends on multiple variables, including the hydraulic resistance, active solute flux, cell length, water permeability, solute transportation and diffusion, and the interaction between actin-network and the cytosol (Eq. 22). For example, passive processes such as solute diffusion dissipate the energy generated from the active solute pumping and decrease the stall force. Figure 1H shows the contour of the stall pressure as an increasing function of the active solute flux, $J_{c,\text{active}}^{\text{f}}$ , and the coefficient of extracellular hydraulic resistance, *d*_*g*_. From the contour and also Eq. 22 we can see that the stall force is a nonlinear function in the coefficient of extracellular hydraulic resistance.

### 3.3 Analysis on the Power Generated by a Migrating Cell

The linear analysis of cell migration shows the reactive forces for actin- and water-driven cell migration. These reactive forces responsible for propelling cell migration are actually dissipative, meaning that these are not the propelling forces that generate the energy for cell migration. Below we analyze the original energy source of cell migration.

To obtain free-energy identities for the one-dimensional steady-state model, we multiply Eq. 1 by *v*_*c*_, Eq. 6 by *v*_*n*_, and Eq. 12 by ln *c*, and integrate the sum over the domain. The power per unit cross-sectional area produced by the cell is found to be

$$\begin{array}{ll} I_{\text{cell}}= & \underset{I_{\text{cell, actomyosin}}}{\underset{︸}{{\left.\left(\frac{de_{n}}{d\theta_{n}}+\frac{\sigma_{a}}{\theta_{n}}\right)\right|}_{0}^{L}J_{\text{actin}}^{\text{f}}+\int_{0}^{L}\sigma_{a}\frac{\partial v_{n}}{\partial x}dx}}\\ & +\underset{I_{\text{cell, solute}}}{\underset{︸}{RT\ln\left(\frac{c{|}_{x=L^{-}}}{c{|}_{x=L^{+}}}\right)J_{c,\text{active}}^{\text{f}}+RT\ln\left(\frac{c{|}_{x=0^{+}}}{c{|}_{x=0^{-}}}\right)J_{c,\text{active}}^{\text{b}}}}, \end{array}$$ (23)

where *e*_*n*_ is the energy density associated with the passive actin network pressure *σ*_*n*_ and satisfies

$$\theta_{n}^{2}\frac{d}{d\theta_{n}}\left(\frac{e_{n}}{\theta_{n}}\right)=\sigma_{n}\left(\theta_{n}\right),\;\text{with}\;{\left.\frac{de_{n}}{d\theta_{n}}\right|}_{\theta_{n}=\theta_{n,*}}=0.$$ (24)

**FIGURE 2:**
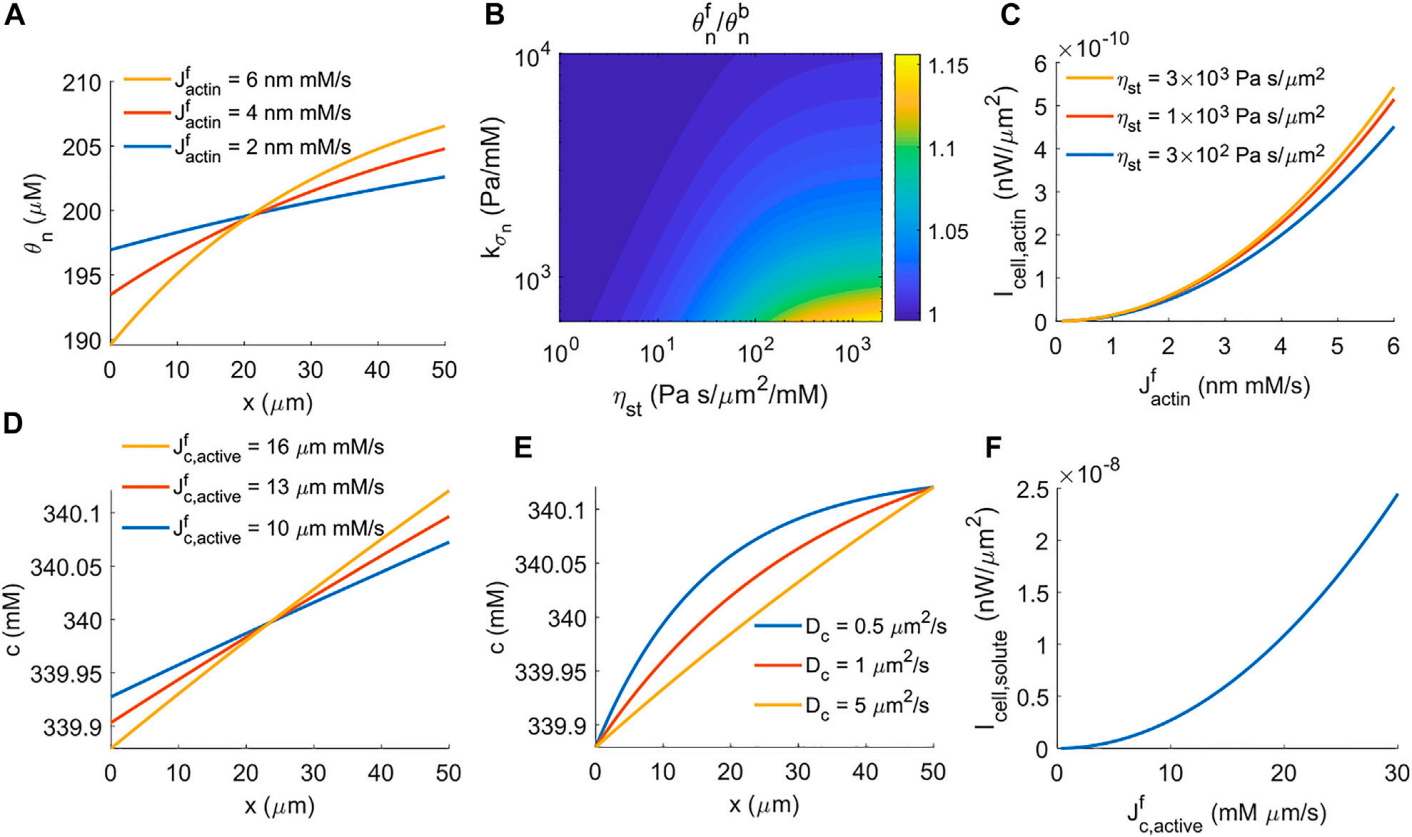
Energy generated by cells under actin-driven and water-driven cell migration. **(A)** Spatial distribution of F-actin concentration for different rates of actin polymerization. **(B)** The contour of the F-actin concentration ratio at the front and back of the cell, $\theta_{n}^{\text{f}}/\theta_{n}^{\text{b}}$ , as a function of the actin pressure coefficient and focal adhesion strength. $\theta_{n}^{\text{f}}=\theta_{n}{|}_{x=L^{-}}$ and $\theta_{n}^{\text{b}}=\theta_{n}{|}_{x=0^{+}}$ . **(C)** Power generation from the actin network as a function of the rate of actin polymerization, $J_{\text{actin}}^{\text{f}}$ , for different strengths of focal adhesion. **(D)** Spatial distribution of solute concentration for different rates of active solute flux. **(E)** Spatial distribution of solute concentration for different coefficients of diffusion. **(F)** Power generation from the active solute pumping as a function of the rate of active solute flux, $J_{\text{c, active}}^{\text{f}}$ .

The first two terms in Eq. 23 are the power generated by the actin polymerization and cytoskeletal stress, whereas the last two terms are the power generated by active solute pumping. These energies originally come from biomolecular processes such as ATP hydrolysis. For a linear constitutive relation for the passive F-actin pressure, i.e., $\sigma_{n}=k_{\sigma_{n}}\theta_{n}$ , the energy density is $e_{n}=k_{\sigma_{n}}\theta_{n}\left[\ln\left(\theta_{n}/\theta_{n,*}\right)-1\right]$ , which is a convex function of *θ*_*n*_. When the active stress from myosin, *σ*_*a*_, is assumed to be zero, the power generation from the actin network reduces to

$$I_{\text{cell, actin}}={\left.\left(\frac{de_{n}}{d\theta_{n}}\right)\right|}_{0}^{L}J_{\text{actin}}^{\text{f}}=k_{\sigma_{n}}\ln\left(\frac{\theta_{n}{|}_{x=L^{-}}}{\theta_{n}{|}_{x=0^{+}}}\right)J_{\text{actin}}^{\text{f}},$$ (25)

which suggests that the work done by actin polymerization against the pressure in the actin network provides the power for actin-driven cell migration. Since $k_{\sigma_{n}}$ is a constant, the power generated by actin is mainly modulated by the rate of actin polymerization and the ratio of F-actin concentration at the front and back ends of the cell, i.e., $\left(\theta_{n}{|}_{x=L^{-}}\right)/\left(\theta_{n}{|}_{x=0^{+}}\right)$ . The spatial profile of the F-actin concentration depends on multiple factors. For example, increasing the rate of actin polymerization increases the polarization of F-actin (Figure 2A). Since actin polymerization happens at the front of the cell, the F-actin concentration will polarize towards the front. We can use the ratio of the F-actin concentration at the front to the back of the cell to indicate the level of F-actin polarization. In addition to the rate of actin polymerization, the ratio increases with increasing strength of focal adhesion, *η*_st_, or decreasing coefficient of actin pressure, $k_{\sigma_{n}}$ (Figure 2B). This is because the effective actin relaxation or diffusion constant is given by $k_{\sigma_{n}}/\eta_{\text{s}t}$ . Reduced F-actin relaxation will lead to an enhanced polarized distribution. As a result, the power generated from the actin network also increases with increasing focal adhesion strength or decreasing actin pressure coefficient (Eq. 25). Since a high rate of actin polymerization increases the front-to-back ratio of F-actin concentration (Figure 2A), the power generated from the actin network thus depends nonlinearly on actin polymerization (Eq. 25, Figure 2C).

In this work we let the extracellular solute concentration be uniform, i.e., $c{|}_{x=0^{-}}=c{|}_{x=L^{+}}$ , and have prescribed $J_{c,\text{active}}^{\text{b}}=-J_{c,\text{active}}^{\text{f}}$ . Therefore, the power generated by active solute pumping reduces to

$$I_{\text{cell, solute}}=RT\ln\left(\frac{c{|}_{x=L^{-}}}{c{|}_{x=0^{+}}}\right)J_{c,\text{active}}^{\text{f}},$$ (26)

which suggests that the work done by active solute pumping against the intracellular solute concentration difference provides the power for water-driven cell migration. In the model, the active solute influx happens at the front of the cell, and thus the intracellular solute concentration is higher at the front than that at the back. Increasing the rate of active solute flux increases the front-to-back ratio of the solution concentration (Figure 2D). The coefficient of solute diffusion, *D*_*c*_, modulates the profile of intracellular solute but does not change the front-to-back concentration ratio (Figure 2E). Similar to the power generated by actin polymerization (Figure 2C), the power generated by active solute pumping is nonlinear in the rate of active solute flux (Figure 2F) because the front-to-back solute concentration ratio depends on the flux as well (Eq. 26, Figure 2D). This power barely depends on the coefficient of the extracellular hydraulic resistance.

## 4 Model Predictions on Mechanosensitivity and Dynamics

Below we will use our model to predict the cell dynamics that are not covered in linear analysis. We will also show the significant difference on the velocity-focal adhesion relation when G-actin is included.

### 4.1 Cell Power Generation Is Mechanosensitive

Cell migration is known to be mechanosensitive. For example, cells tend to migrate towards locations with high substrate stiffness, known as durotaxis (Sunyer and Trepat, 2020); or migrate towards directions with low hydraulic resistance, known as barotaxis (Prentice-Mott et al., 2013; Zhao et al., 2019; Li et al., 2020). Below we will use the mathematical model to show that the actin-driven cell power generation is also mechano-sensitive, i.e., the power generation is different with and without external pressure.

**FIGURE 3:**
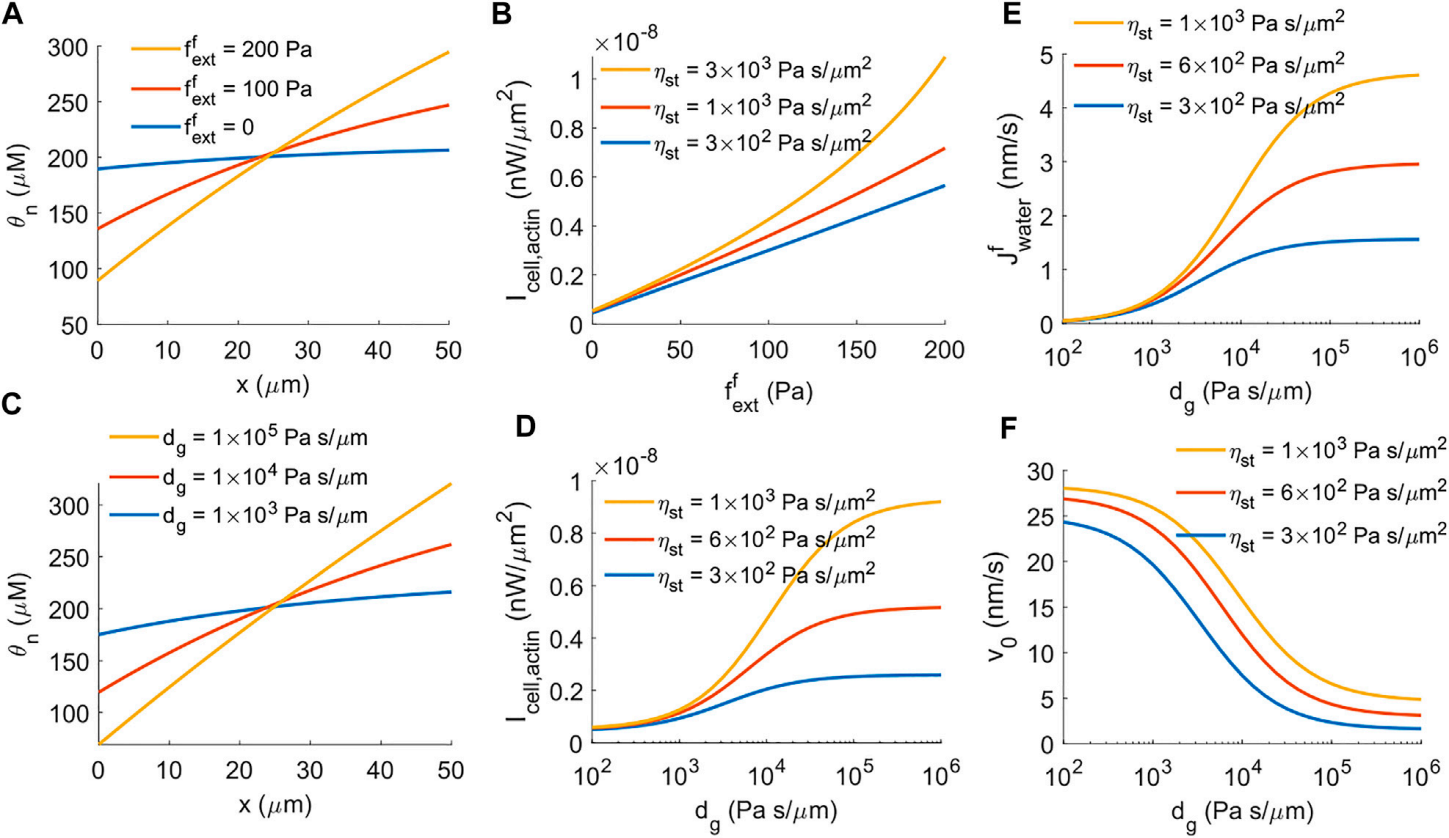
The impact of external force on the energy generation of actin-driven cell migration. **(A)** Spatial distribution of F-actin concentration for different magnitudes of external force per unit cross-sectional area, $J_{\text{ext}}^{\text{f}}$ . **(B)** Power generation from the actin network as a function of the external force per unit cross-sectional area, $J_{\text{ext}}^{\text{f}}$ , for different strengths of focal adhesion, *η*_st_. **(C)** Spatial distribution of F-actin concentration for different coefficients of external hydraulic resistance, *d*_*g*_. **(D)** Power generation from the actin network as a function of the coefficient of external hydraulic resistance, *d*_*g*_, for different strengths of focal adhesion, *η*_st_. **(E)** Under actin-driven cell migration, water flux across the cell as a function of the coefficient of external hydraulic resistance, *d*_*g*_, for different strengths of focal adhesion, *η*_st_. **(F)** The velocity of actin-driven cell migration as a function of the coefficient of external hydraulic resistance, *d*_*g*_, for different strengths of focal adhesion, *η*_st_.

Given a constant rate of actin polymerization and a coefficient of passive F-actin stress, Eq. 25 shows that the front-to-back F-actin concentration ratio determines the power generation from the actin network. We observe that in the presence of an external force, the distribution of F-actin is significantly polarized (Figure 3A). This is because the external force reduces the cell migration velocity, *v*_0_. On the other hand, the boundary condition for the conservation of F-actin at the front of the cell is $\theta_{n}\left(v_{n}-v_{0}\right)=-J_{\text{actin}}^{\text{f}}$ , which gives $\theta_{n}=J_{\text{actin}}^{\text{f}}/\left(v_{0}-v_{n}\right)$ at *x* = *L* ^−^. Therefore, decreasing *v*_0_ leads to increasing $\theta_{n}{|}_{x=L^{-}}$ . The passive pressure in the actin network increases with F-actin concentration, meaning it requires more work for newly polymerized F-actin to push against the existing F-actin towards the interior of the cell. As a result, the power generation from the actin network increases significantly (by two orders of magnitude) in the presence of the external force (Figure 3B) compare to the case without the external force (Figure 1C), even if the rate of actin polymerization remains the same.

Besides applied external pressure or forces from physical barriers, extracellular hydrostatic pressure also provides mechanical cues for cell migration. The model predicts that the distribution of F-actin is more polarized when there is elevated external hydraulic resistance in front of the cell (Figure 3C). The power generation from actin polymerization increases accordingly with increasing hydraulic resistance (Figure 3D), as with a prescribed external force (Figure 3B). One main difference between external force and hydraulic resistance is that under hydraulic resistance, water flux across the cell membrane is induced even if there is no active solute pumping (Eq. 19). The model predicts increased water flux into the cell from the front with increasing extracellular hydraulic resistance (Figure 3E). As a result, unlike the external force which is able to stall cell migration (Figure 1E), external hydraulic resistance does not fully stall the cell migration (Figure 3F), where the residual cell velocity comes from water-induced actin-driven cell migration (Eq. 19, the second term in the front of $J_{\text{actin}}^{\text{f}}$ ). This residual velocity gives rise to the plateau of the power generated from actin polymerization at the high limit of hydraulic resistance (Figure 3D).

These results indicate that the power generated by actin polymerization is mechanosensitive such that higher external pressure against cell migration increases the power generation by the cell. This result has implications for how cells can overcome external barriers through enhanced power generation mechanisms.

### 4.2 Actin Dynamics Determines the Biphasic Response on Focal Adhesion

**FIGURE 4:**
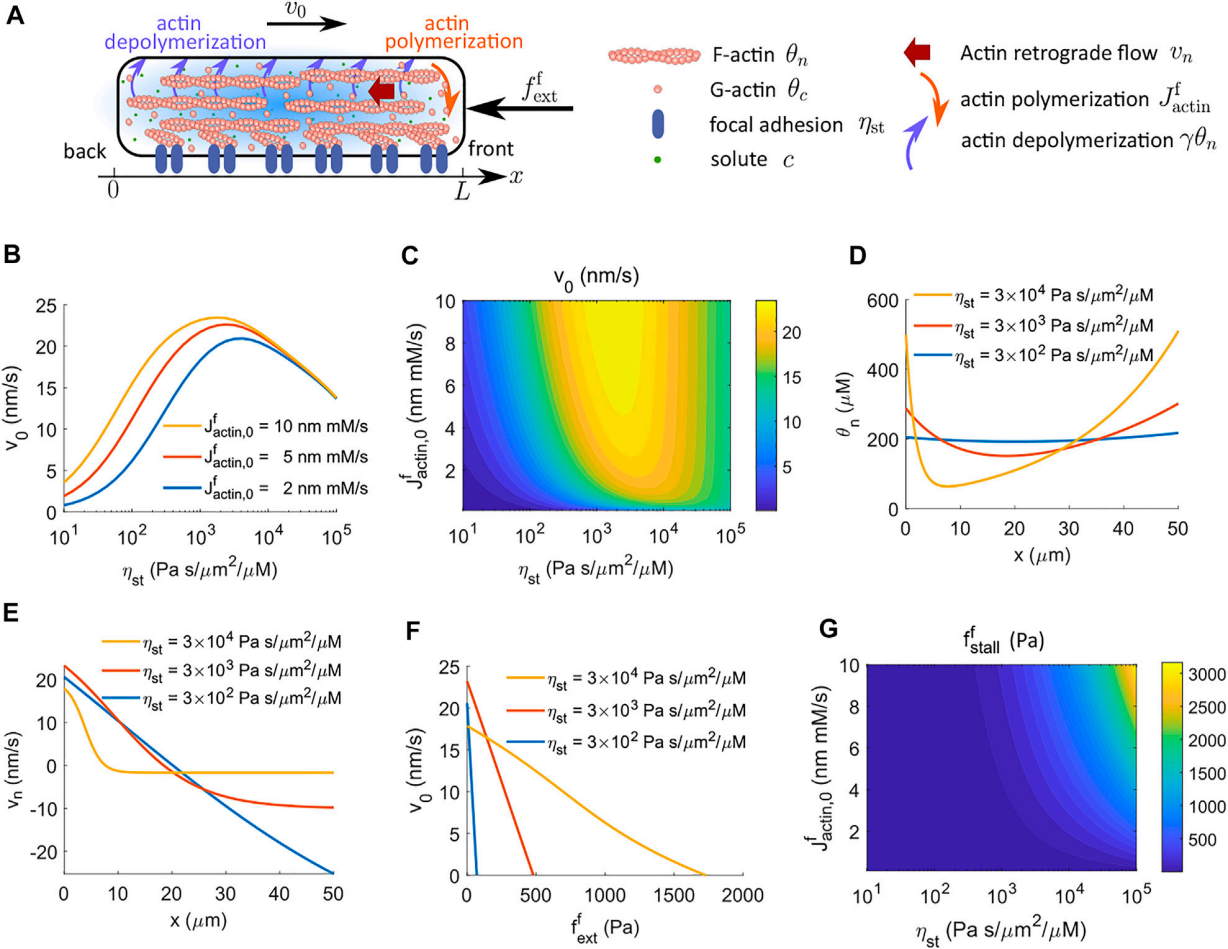
Cell velocity and effective force generation under actin-driven cell migration when actin depolymerization happens throughout the cytoplasm. The model includes G-actin. **(A)** Schematics of actin-driven cell migration where actin polymerization happens at the front end of the cell while depolymerization occurs throughout the entire cell. **(B)** Model prediction of the velocity of cell migration as a function of the strength of focal adhesion, *η*_st_, for different rates of actin polymerization at the front end of the cell. The cell velocity is biphasic in *η*_st_. **(C)** The contour of the predicted cell velocity, *v*_0_, as a function of the coefficient of actin polymerization, $J_{\text{actin},0}^{\text{f}}$ , and the strength of focal adhesion, *η*_st_. **(D)** The spatial distribution of F-actin concentration for different strengths of focal adhesion, *η*_st_. **(E)** The spatial profile of actin retrograde flow, *v*_*n*_, for different strengths of focal adhesion, *η*_st_. **(F)** Predicted actin-driven cell velocity as a function of the external force per unit area, $f_{\text{ext}}^{\text{f}}$ , for different strengths of focal adhesion, *η*_st_. **(G)** The contour of the stall force per unit area for cell migration as a function of the coefficient of actin polymerization, $J_{\text{actin},0}^{\text{f}}$ , and the strength of focal adhesion, *η*_st_.

Cells *in vivo* experience different biomolecular and biophysical environments, which can affect how cells modulate spatial actin dynamics. We have discussed an actin-driven case in an early model where actin polymerization and depolymerization happen at the front and back ends of the cell, respectively (Figure 1A). Here we discuss a different actin dynamics model, with the inclusion of G-actin, where polymerization still happens at the front of the cell, but depolymerization occurs throughout the cytoplasm (Figure 4A; Section 2.2.2).

When actin depolymerization occurs throughout the cytoplasm, the model predicts that the cell velocity is biphasic in the strength of focal adhesion, *η*_s*t*_, meaning that the cell needs sufficient forces from focal adhesion to migrate efficiently, but excessive forces slow down migration (Figure 4B) (DiMilla et al., 1993; Palecek et al., 1997; Gupton and Waterman-Storer, 2006; Gardel et al., 2008; Kim and Wirtz, 2013). A contour of cell velocity as a function of the coefficient of actin polymerization, $J_{\text{actin},0}^{\text{f}}$ , and the strength of focal adhesion, *η*_st_, provides a clear overall picture on the biphasic behavior (Figure 4C). This result is different from the model where actin polymerization and depolymerization, respectively, happen at the front and back ends of the cell (Figure 1C; Section 2.2.1).

The physical interpretation underlying this biphasic cell velocity in focal adhesion comes from the distribution of F-actin and the magnitude of actin retrograde flow. When actin depolymerization happens only at the back end of the cell, the F-actin concentration is monotonic in space (Figure 2A). The amount of spatial variation is small compared to the average F-actin concentration (Figures 2A,B). In addition, the conservation equation *d* (*θ*_*n*_ *v*_*n*_)/*dx* = 0 shows that *θ*_*n*_ *v*_*n*_ is a constant in space, meaning that the reactive force from focal adhesion, *η*_st_ *θ*_*n*_ *v*_*n*_, is also constant in space and increases with the strength of focal adhesion. In contrast, when actin depolymerization occurs throughout the cell cytoplasm, the F-actin concentration is high at the two ends of the cell and is low in the interior of the cell (Figure 2D). The amount of spatial variation of F-actin concentration increases with the strength of focal adhesion. The model also suggests that the effective spatial region of F-actin shrinks towards the two ends of the cell as the strength of focal adhesion increases. Meanwhile, with high focal adhesion strength, the actin retrograde flow is close to zero at the front of the cell (Figure 2E). This small backward F-actin retrograde flow provides limited reactive force from focal adhesion for cell migration. At the back end of the cell, the actin moves forward with the moving cell. This forward motion creates a reactive force from focal adhesion that resists cell migration. The combined F-actin concentration and flow pattern reduce cell velocity at high focal adhesion strength.

The cell velocity decreases progressively with increasing amplitude of the external resistive force per unit area applied to the front of the cell (Figures 2A,F). Interestingly, the stall force per unit area still increases monotonically with the coefficient of actin polymerization, $J_{\text{actin},0}^{\text{f}}$ , and the strength of focal adhesion, *η*_st_, regardless of the biphasic velocity of cell velocity (Figure 2G). This model prediction suggests that the stall force is not solely determined by the cell velocity but depends on the biomolecular processes such as actin polymerization and forces from integrin proteins. As the strength of focal adhesion increases, the actin network adheres more strongly to the focal adhesion, which requires larger external forces to counteract this adhesion irrespective of the cell velocity. At the peak of the biphasic velocity where *η*_st_ is on the order of 10^3^ Pa s/μm^2^/μM, the stall force per unit area is on the order of 1 kPa. This magnitude also corresponds to a stall force on the order of 30–50 pN, as observed in experiments (Oliver et al., 1995; Prass et al., 2006).

### 4.3 Cell Shape Affects the Stall Force

**FIGURE 5:**
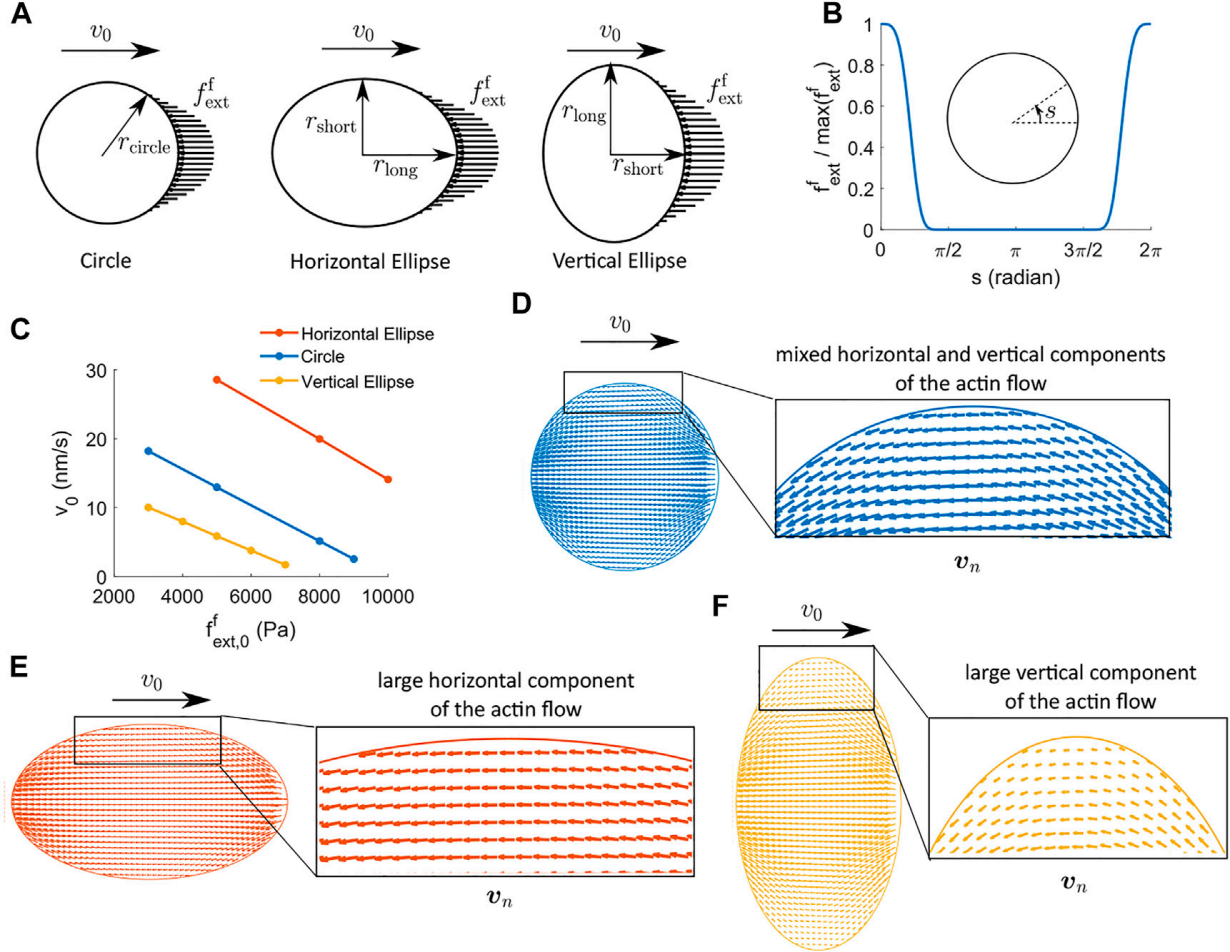
Two-dimensional cell velocity and effective force generation under actin-driven cell migration when actin polymerization happens at the front regime of the cell and depolymerization happens at the back region of the cell. **(A)** Schematics of the external force per unit area applied to the front region of the cells. We use three cell shapes: circle, horizontal ellipse, and vertical ellipse. The three cells have the same areas. *r*_circle_ = 14.92 μm, *r*_long_ = *r*_circle_/*a*, and *r*_short_ = *ar*_circle_, where *a* = 0.75 is a parameter for the aspect ratio of the ellipse. **(B)** Profile of the external force. $f_{\text{ext}}^{\text{f}}/\max\left(f_{\text{ext}}^{\text{f}}\right)=g\left(s\right)$ is the shape profile of the external force applied at the front regime of the cell. *α*_ext_ = 0.2*π*. **(C)** Actin-driven cell migration as a function of the magnitude of the external force, $f_{\text{ext},0}^{\text{f}}$ , for three different cell shapes. **(D–F)** The spatial profile of the actin network velocity, ***v***_*n*_, in the absence of external forces for the circle, horizontal ellipse, and the vertical ellipse cells, respectively. The actin velocity in the horizontal ellipse-shaped cell has a relatively large component along the direction of migration. The actin velocity in the vertical ellipse-shaped cell has a relatively large component perpendicular to the direction of cell migration.

In this section, we use a two-dimensional implementation to study how the morphology of a cell affects cell force generation. We consider three cell shapes: a circle, a horizontal ellipse where the cell elongates along the direction of migration, and a vertical ellipse where the cell elongates along the transverse direction of migration (Figure 5A). The three shapes have the same area, i.e., $r_{\text{long}}r_{\text{short}}=r_{\text{circle}}^{2}=r^{2}$ , so that the energy dissipation from intracellular processes in the three cells occurs in spaces of the same size. The region of actin polymerization, depolymerization, active solute flux, and the external force is chosen such that the line integral of the respective quantities remains the same for the three cells. By keeping all these forcing contributions the same, we are able to investigate whether the shape of a cell plays a role in the effective force output during cell migration.

The external force per unit area is applied at the front region of the cell (Figure 5A) with a maximum amplitude $f_{\text{ext},0}^{\text{f}}$ and a spatial profile *g*(*s*) given by

$$f_{\text{ext}}^{\text{f}}\left(s\right)=f_{\text{ext},0}^{\text{f}}g\left(s\right),\;g\left(s\right)=\exp\left(-\frac{s^{4}}{2\alpha_{\text{ext}}^{4}}\right)+\exp\left(-\frac{{\left(s-2\pi \right)}^{4}}{2\alpha_{\text{ext}}^{4}}\right),$$ (27)

where *s* describes the angular coordinate in radians along the cell boundary running in the counterclockwise direction, *α*_ext_ is the half span angle of the external force, and *g*(*s*) is a shape profile of the external force (Figure 5B). The distribution of actin polymerization, depolymerization, and active solute flux is implemented in a similar way. Here we focus on the case where actin depolymerization happens at the back of the cell, not throughout the cytoplasm.

The model predicts several interesting results. Under the same strength and distribution of actin (de)polymerization for all cells, the baseline cell migration velocity, i.e., the velocity without any external forces, is higher for the horizontally elongated cell compared to the vertically elongated cell by more than 20 nm/s (Figure 5C). The difference in the baseline velocity in the three cells of different shapes comes from the spatial distribution of the actin flow (Figures 5D–F). The horizontally elongated elliptical cell has a large component of the actin flow along the direction of cell migration, which gives rise to non-trivial reactive force from the focal adhesion. The vertically elongated elliptical cell, on the other hand, has a significant component of the actin flow perpendicular to the direction of cell migration, which does not generate substantial reactive force from the focal adhesion. The circular-shaped cell has mixed components of the actin flow, and thus its baseline velocity is between the two elliptical cells. The two-dimensional model confirms the importance of the actin flow in reactive force generation discussed in the one-dimensional model.

The cell velocity decreases almost linearly as a function of the external force, similar to the one-dimensional results (Figures 1E,F). The sensitivity of the cell velocity to the external force, i.e., the slope of the *v*_0_ versus $f_{\text{ext}}^{\text{f}}$ curve, is very similar for the three cell shapes. Therefore, the stall force per unit area is scaled by the baseline cell velocity and is thus cell-shape dependent (Figure 5C). For a circular cell, the predicted stall force per unit area, $f_{\text{ext},0}^{\text{f}}$ , is on the order of 10 kPa, the same as the pressure against actin on mouse 3T3 cells (Abraham et al., 1999). The effective stall force, $F_{\text{ext}}^{\text{f}}$ , can be estimated from the stall pressure, $f_{\text{ext}}^{\text{f}}$ , through an effective area of force contact, *A*, i.e., $F_{\text{ext}}^{\text{f}}=Af_{\text{ext},0}^{\text{f}}$ . We will use the circular cell as an example to obtain the effective area of force contact. The external force exerts onto the lamellipodia, which on average has a thickness of ∼200 nm for 3T3 cells and keratocytes (Abraham et al., 1999; Laurent et al., 2005). We, therefore, approximate the effective height of a cell as *H* = 200 nm. Since the external force is symmetric with respect to the *x*-axis, the area is thus $A=2H\int_{0}^{\pi }rg\left(s\right)ds=2Hr\int_{0}^{\pi }g\left(s\right)ds$ . Based on the spatial distribution of the external force and the radius of the cell, the contact area of the force is on the order of *A* = 4 μm^2^. Therefore, the corresponding stall force is on the order of 40 nN, which is also consistent with experimental measurement (Oliver et al., 1995; Prass et al., 2006).

## 5 Conclusion and Discussion

In this work, we use a multi-modular mathematical framework to quantify the effective force generated from actin-driven and water-driven cell migration. The results show that the effective force generated by actin-drive cell migration is proportional to the rate of actin polymerization and the strength of focal adhesion; the energy source comes from the actin polymerization against the actin network pressure. The effective force generated by water-driven cell migration is proportional to the rate of active solute flux and the coefficient of external hydraulic resistance; the energy sources come from the active solute pumping against the solute concentration gradient. We also studied the differences in cell velocity and the similarity of cell force generation for models with and without G-actin. In particular, our model demonstrates that the presence of G-actin can lead to a biphasic cell velocity in the strength of focal adhesion. The model further predicts that the spatial distribution of the actin network is mechanosensitive. The cell velocity and effective force generation also depend on the cell shape through the intracellular actin flow field. Our prediction provides insights into the force production in biological processes.

The biphasic dependence of cell velocity on the strength of focal adhesion has been observed from experiments (DiMilla et al., 1993; Palecek et al., 1997; Gupton and Waterman-Storer, 2006; Gardel et al., 2008; Kim and Wirtz, 2013). Models that use spring-like elements have also been successful in predicting the biphasic behavior (DiMilla et al., 1991). We use a multi-modular model that does not involve spring-like elements. The model suggests that the presence of biphasic velocity can also depend on how actin depolymerization occurs. When actin depolymerization happens only at the back end of the cell, the reactive force from focal adhesion is uniformly distributed within the cell, leading to monotonically increasing cell velocity with the strength of focal adhesion. When actin depolymerization happens throughout the cytoplasm, the F-actin moves towards the two ends of the cell, and the effective spatial region where focal adhesion provides a positive reactive force for cell migration shrinks with increasing strength of focal adhesion. These lead to experimentally-observed biphasic cell velocity with focal adhesion. In addition, given that the persistence length of F-actin is about 16 μm (Gittes et al., 1993; Ott et al., 1993), actin filaments are bundled or cross-linked within the cell, which leads to actin depolymerization throughout the cytoplasm. Taken together, a model that includes actin depolymerization throughout the cytoplasm may be more desirable.

The model predicts that water-driven cell migration requires a certain level of hydraulic resistance. This requirement is consistent with the experimental observation that water-driven cell migration happens when cells are confined in flow-limited space (Stroka et al., 2014). Here we discuss the range of the coefficient of extracellular hydraulic resistance that is physiologically relevant. When cells migrate in open, two-dimensional substrate, the hydraulic resistance is negligible becuase the sourrounding fluid can flow freely. When cells migrate in confined, one-dimensional channels, the hydraulic resistance is approximated as *d*_*g*_ = 12*μℓ*/*b* ^2^ (Li and Sun 2018), where *μ* is the extracellular fluid viscosity, *ℓ* is the channel length, and *b* is the smallest dimension of the cross-sectional area of the channel. If *μ* = 10^–2^ Pa⋅s, *ℓ* = 10^2^–10^3^ μm, and *b* = 3 μm, then *d*_*g*_ ranges from 1 Pa⋅s/μm to 10 Pa⋅s/μm. When cells migrate in infinite, three-dimensional collagen matrices, the hydraulic resistance is approximated as *d*_*g*_ = *μw*/2*κ* (Li and Sun, 2018), where *w* is the characteristic cross-sectional length of the protrusion and *κ* is the collagen permeability which may range from 10^–4^ μm^2^–10^2^ μm^2^ (Vennat et al., 2010; Polachecka et al., 2011; Gjorevski and Nelson 2012; Jansen et al., 2018; Maity et al., 2019). If we take *w* = 10 μm, then *d*_*g*_ ranges from 10^–3^ Pa⋅s/μm to 10^3^ Pa⋅s/μm. This range is within the regime of *d*_*g*_ where water contribution to cell migration is non-trivial, based on our model prediction (Figure 1D).

The model estimates that it costs about two orders of magnitude more power for cells to generate water-driven migration than actin-driven migration (Figures 2C,F) to achieve a similar cell velocity. This difference means that active solute pumping against the solute concentration gradient (Eq. 26) costs more energy than that of actin polymerization against the actin network (Eq. 25), i.e., the chemical work is larger than the mechanical work. The ATP consumption associated with active ion pumps is about two to three orders of magnitude higher than the ATP consumption associated with actin dynamics (Li et al., 2019). We can therefore conclude that water-driven cell migration requires more energy input. Interestingly, the model predicts that the stall force for water-driven cell migration is lower than that for actin-driven cell migration (Figures 1G,H). This prediction shows that the effective cell force output is not necessarily proportional to the cell energy input. This is because the chemical work input for water-driven migration mostly dissipates through chemical processes such as passive solute flux and diffusion, and only a fraction dissipates through mechanical processes such as hydraulic resistance. In this model, we used a relatively low strength of focal adhesion for water-driven cell migration compared to the actin-driven cell migration (Liu et al., 2015; Paluch et al., 2016). If the strength of focal adhesion remains the same for two migratory mechanisms, the hydraulic resistance for water-driven cell migration needs to increase to overcome the resistance from focal adhesion. In this case, the effective cell force output from water-driven cell migration will be higher than the force from actin-driven migration (Li et al., 2019).

In the model, we have assumed that the rate of actin polymerization, $J_{\text{actin}}^{\text{f}}$ , does not change with the external force. Experimentally it has been found that the rate of actin polymerization remains constant when the external force per unit area ranges from 150 to 500 Pa (Parekh et al., 2005). The polymerization rate decreases when the external force per unit area goes beyond 500 Pa and stops at 1 kPa (Parekh et al., 2005). If we incorporate into the model the dependence of the actin polymerization rate on the magnitude of the external force, the cell velocity-external force relation (Figures 1E, Figure 4F) will bend downwards in a similar manner as that reported in Prass et al. (2006). In this case, the predicted stall force will be lower than the current predicted results. Interestingly, the model predicts that an external force will change the F-actin distribution and thus leads to a higher cell energy input (Figures 3C,D) compared to the case without an external force. This difference suggests that cells will reorganize their cytoskeleton structure upon external mechanical input. Experimentally, it has indeed been observed that the actin network can further polarize towards the front of the cell when cells experience high external hydrostatic pressure (Zhao et al., 2019; Zhao et al., 2021).

## Data Availability

The original contributions presented in the study are included in the article/Supplementary Material, further inquiries can be directed to the corresponding author.
